# Empowerment: Equipping Patients and Caregivers With the Confidence to Act

**DOI:** 10.1097/jnr.0000000000000753

**Published:** 2026-05-26

**Authors:** Yuan-Mei LIAO

The empowerment approach is a partnership between individuals and healthcare providers that draws on the premise that everyone has the inherent power to lead their own journey (Werbrouck et al., 2018). This approach recognizes that individuals possess competencies and the right to function independently. More than simply granting rights, empowerment ensures the social structures and resources necessary to actualizing these rights are provided, allowing individuals to demonstrate their abilities and exercise effective control over their own lives.

Eïd and Desgrées du Loû’s (2022) review of empowerment-based programs revealed empowerment to be both a process and a goal that is achieved through training, participation, and personalized assistance. With empowerment as the ultimate goal, healthcare providers can enhance perceived self-efficacy in patients and caregivers and support them in self-managing their conditions. Perceived self-efficacy refers to the beliefs of individuals regarding their own ability to perform actions required to deal with prospective situations (Bandura, 1982). Individuals with high perceived self-efficacy tend to view challenges as opportunities rather than problems and to set more ambitious goals and be more self-motivated to achieve them (Warner & Schwarzer, 2020). By approaching challenges with determination and investing greater effort into overcoming obstacles, individuals with high self-efficacy create more opportunities to experience mastery (Bandura, 1977). Self-efficacy is the central mechanism powering effective self-management, a function that helps regulate chronic condition control, reduce caregiver burden, enhance health outcomes, and optimize expense management (Polsook et al., 2024).

Reflecting the increasing engagement of family members and the incorporation of digital solutions in healthcare, the two articles that address empowerment in this issue investigate the role of self-efficacy and offer insights into empowering both patients and caregivers to achieve sustainable outcomes. One of these, a longitudinal study by Lee et al.'s (2026), highlights a critical advancement in chronic disease management. By exploring the relationship between eHealth literacy and self-management among patients with type 2 diabetes, the authors find that having digital knowledge alone is not sufficient and that self-efficacy fully mediates the effect of eHealth literacy on self-management. Thus, health professionals must go beyond teaching patients how to use technical tools and focus on building confidence to act by enhancing their self-efficacy to effectively bridge the gap between knowledge and action. Family caregivers of nursing home residents often experience many multifaceted challenges, making empowered caregivers essential partners in the long-term care ecosystem. Hsiao et al.'s (2026) evaluation of a nurse-led, web-based communication education program highlights how digital interventions can empower the family caregivers of nursing home residents. As the communication self-efficacy of caregivers increases, they tend to exhibit greater initiative and management skills throughout the caregiving journey. The communication education program addressed in this study equips caregivers to engage in effective interactions, leading to improved family-centered care.

Nurses play a vital role in fostering the ability of patients and caregivers to engage actively in the situations they encounter. Empowerment is essential to maximizing the effectiveness of interventions in managing chronic conditions and navigating the complexities of healthcare. The ability of empowerment-based interventions, including those supporting dyadic coping and family-centered care, to improve study-targeted patient and caregiver outcomes is supported by robust evidence (Araújo et al., 2026; Stepanian et al., 2023). Also, research evidence supports the use of innovative interventions and interactive digital tools in improving patient and family empowerment (Mulyana et al., 2023; Tuominen et al., 2024). By intentionally cultivating self-efficacy, both patients and caregivers can gain the confidence necessary to manage multifaceted challenges and take charge of their life journeys. We hope these studies inspire readers to incorporate empowerment-based strategies into their clinical practices and research.

Copyright © 2026 The Author Copyright (s). Published by Wolters Kluwer Health, Inc. on behalf of the Taiwan Nurses Association.

This is an open access article distributed under the Creative Commons Attribution License 4.0 (CCBY), which permits unrestricted use, distribution, and reproduction in any medium, provided the original work is properly cited.
